# Pore Formation Process of Porous Ti_3_SiC_2_ Fabricated by Reactive Sintering

**DOI:** 10.3390/ma10020163

**Published:** 2017-02-10

**Authors:** Huibin Zhang, Xinli Liu, Yao Jiang

**Affiliations:** 1 State Key Laboratory of Powder Metallurgy, Central South University, Changsha 410083, China; zhanghb@csu.edu.cn (H.Z.); jiangyao@csu.edu.cn (Y.J.); 2 School of Metallurgy and Environment, Central South University, Changsha 410083, China

**Keywords:** Ti_3_SiC_2_, metal ceramic, porous, reaction procedure, pore formation process

## Abstract

Porous Ti_3_SiC_2_ was fabricated with high purity, 99.4 vol %, through reactive sintering of titanium hydride (TiH_2_), silicon (Si) and graphite (C) elemental powders. The reaction procedures and the pore structure evolution during the sintering process were systematically studied by X-ray diffraction (XRD) and scanning electron microscope (SEM). Our results show that the formation of Ti_3_SiC_2_ from TiH_2_/Si/C powders experienced the following steps: firstly, TiH_2_ decomposed into Ti; secondly, TiC and Ti_5_Si_3_ intermediate phases were generated; finally, Ti_3_SiC_2_ was produced through the reaction of TiC, Ti_5_Si_3_ and Si. The pores formed in the synthesis procedure of porous Ti_3_SiC_2_ ceramics are derived from the following aspects: interstitial pores left during the pressing procedure; pores formed because of the TiH_2_ decomposition; pores formed through the reactions between Ti and Si and Ti and C powders; and the pores produced accompanying the final phase synthesized during the high temperature sintering process.

## 1. Introduction

Porous ceramics, which have outstanding properties, such as excellent corrosion resistance, good structural stability and high hardness, are extensively used in various fields, such as filtration, catalysis and environmental protection [1,2,3,4]. Traditionally, ceramics have intrinsic disadvantages, for instance high brittleness, poor machinability and low conductivity, which limit their potential applications [5,6]. Recently, layered ternary metal ceramics, with the general formula M_N + 1_AX_N_ (N = 1, 2 or 3), where M is a transition metal, A is an A-group (mostly IIIA and VIA) element and X is either C or N, are investigated intensively due to their excellent properties [7]. Ti_3_SiC_2_ is a representative of this material. It has a hexagonal crystalline lattice with typical laminate characteristic [7] and is anisotropic and metallic-covalent-ionic bonding in nature [8]. Owning to the features of the crystal lattice and bond structures, Ti_3_SiC_2_ has flexibility, is elastically stiff, has relatively low thermal expansion coefficients and good corrosion resistance as ceramics [7,9]. It should be noted that it also exhibits some metal-like properties, such as ductility and malleability, high machining performance and good damage tolerance [7,10]. Hence, porous Ti_3_SiC_2_ is expected as an ideal porous material applied in different fields that cannot be satisfied by traditional ceramics.

Previous reports on porous Ti_3_SiC_2_ mainly focused on the synthesis of this material by reactive sintering using different elemental powders and its mechanical properties [11,12]. Few investigations were published on pore formation and phase transformation during the synthesizing process. Note that the purity of Ti_3_SiC_2_ also could not be guaranteed in previous works, even for bulk Ti_3_SiC_2_. Recently, we have successfully fabricated porous Ti_3_SiC_2_ [10] and microporous Ti_3_SiC_2_ membranes [13] by powder metallurgy, and the purity is more than 99.0 vol %. The pore structure parameters, including porosity, pore size and distribution, can be controlled by the initial powders, pressing pressure and the sintering process [14]. However, the pore formation mechanism has been only discussed briefly [10]. It is important to understand the pore evolvement procedure accompanying the phase transformation process for the fabrication of porous Ti_3_SiC_2_. Moreover, the systematic research on the formation mechanism of porous Ti_3_SiC_2_ will provide a universal route to the synthesis of porous MAX phases.

In this paper, firstly, we show that porous Ti_3_SiC_2_ with high purity and micrometer-sized pores can be synthesized through reactive synthesis employing TiH_2_, Si and graphite powders as raw materials. Secondly, we utilized techniques, such as DTA, XRD and SEM, to explain how the pores are formed and evolve during the reactive sintering process.

## 2. Experimental Details

### 2.1. Sample Synthesis

Titanium hydride (TiH_2_), silicon (Si) and graphite (C) powders with 99.5 wt % purities were employed as the raw materials to synthesize porous Ti_3_SiC_2_. The particle sizes of TiH_2_, Si and C powders are 38.4 μm, 5.6 μm and 6.2 μm as the median diameters, respectively. The nominal composition is 72.1 wt % TiH_2_-16.3 wt % Si-11.6 wt % C, with the element mole ratio of Ti:Si:C = 3:1.2:2. Excessive silicon powders were added in order to compensate for their loss at high temperature [15] and to improve the purity of the final samples [13]. Raw powders were milled under 100 rpm for 12 h with ethanol as the milling medium. The weight ratio was set to be 3:1 of grinding media to material. After this step, the mixed powders were dried at 60 °C in a vacuum oven to remove ethanol.

Green compacts, with the dimensions of ~φ 30 mm × ~3 mm, were formed by cold-pressing under pressure of 200 MPa. The green compacts were sintered in a vacuum furnace with a Mo heating element under pressure of ~10^−3^ Pa from 500 to 1350 °C and held at every hundred degrees for 1 h to study the reactive synthesis procedure. In order to make the heating rate consistent when the temperature raised from room temperature to 1350 °C, we chose the heating rate of 3 K/min. Samples were finally sintered at 1350 °C for 3 h. Fifty green compacts were primarily put into furnace, and 5 samples were taken out after holding at every hundred degrees from 500 to 1300 °C for volume measurement, phase analysis and pore structure characterization.

### 2.2. Characterization of Samples

The thermal behavior of the green compact was analyzed by differential thermal analysis (DTA: WCT-2A, Tokyo, Japan). The composition of samples was characterized by X-ray diffraction (XRD: Dmax 2500VB, Tokyo, Japan) with a Cu Kα after sintering procedure. Field-emission scanning microscopy (Nova Nano SEM 230, Waltham, MA, USA) was employed to analyze the morphologies of samples sintered at different temperatures. The open porosity of synthesized materials was measured according to the Archimedes method in water. The maximum pore size was tested on a porous material test instrument (FBP-3III, Northwest Institute for Non-Ferrous metal Research, Xi’an, China) using N_2_ as the fluid medium by the bubble point method.

## 3. Results and Discussion

### 3.1. Thermal Behavior of the Green Compact

**Figure 1:**
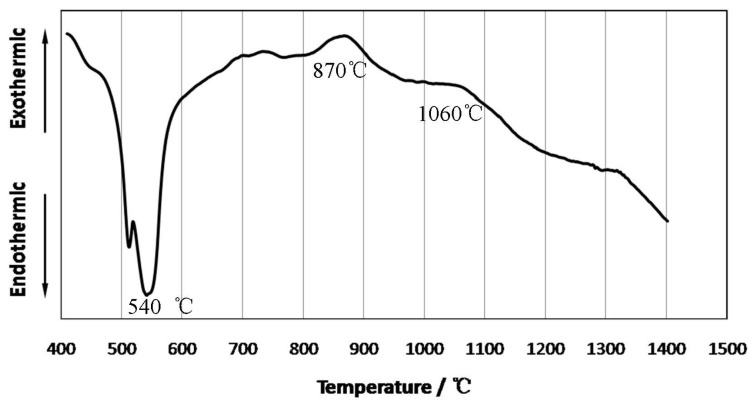
DTA profile of the TiH_2_-Si-C compact in an argon atmosphere with a heating rate of 10 K/min.

Figure 1 shows the DTA profile of the compact with the nominal composition of 72.1 wt % TiH_2_-16.3 wt % Si-11.6 wt % C in an argon atmosphere with the heating rate of 10 K/min. This curve reveals the thermal behavior of the TiH_2_-Si-C green compact, which has an important influence on the reactive synthesis procedure of Ti_3_SiC_2_. We can see an obvious endothermic behavior at the range of 450–650 °C and an intensive endothermic peak at ~540 °C, which corresponds to the decomposition of TiH_2_ [16]. The generation of dehydrogenated Ti is propitious to the subsequent synthesis of the intermediate compound [17,18]. Slopes of the curve gently go upward with a relatively weak exothermic peak at ~870 °C, which can be interpreted as a gradual solid diffusion reaction among Ti, Si and C in the range of 800–950 °C. After that, the curve shows a much weaker exothermic reaction in the range of 1000–1150 °C with the final reaction peak at ~1060 °C. As is commonly known, there is a self-propagation high-temperature synthesis (SHS) phenomenon in the Ti-Si-C system [19]. However, there was no strong reaction during the sintering procedure of the TiH_2_-C-Si system. According to Figure 1, it can be related to Ti from dehydrogenation facilitating the reaction between Ti–X (X = Si or C) elements by decreasing the initial reaction temperature. Ti generated during sintering has higher chemical reaction activity and, hence, a lower requirement regarding the reaction temperature, since it has a clean reaction interface and some distorted crystal lattices because of the decomposition. The decrement of the reaction temperature leads to gentle heat release. As a result, the SHS phenomenon was depressed in our experiments, which can make the reactive synthesis procedure more controllable.

### 3.2. Reaction Process

**Figure 2:**
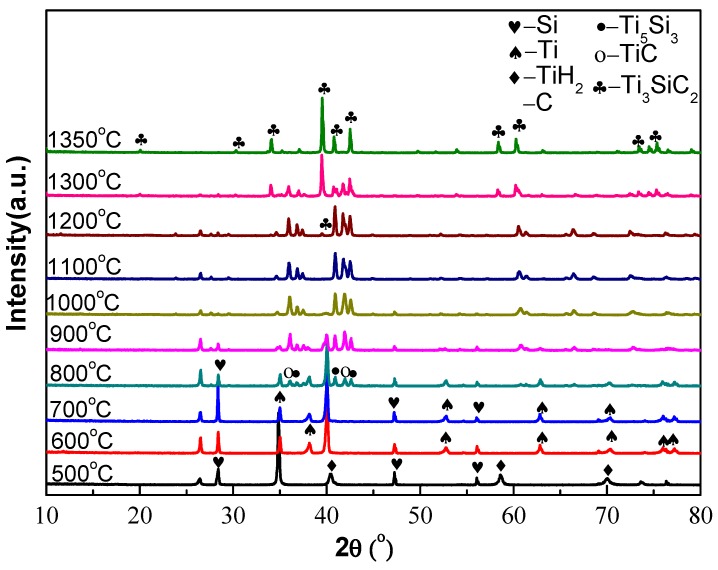
XRD patterns of the compacts under different final sintering temperatures.

In order to figure out the phase transition during the sintering process, the composition of the compacts sintered at different temperatures was analyzed by XRD, shown in Figure 2. For the compact sintered at 500 °C, only pure TiH_2_, Si and C phases were detected, indicating that there was no reaction happened among them. When the sintering temperature increased to 600 °C, there were some changes in the XRD patterns. We can observe that peaks from Ti appear in the XRD pattern of samples sintered at 600 °C. This confirms that TiH_2_ begins to decompose into Ti, which is consistent with the previous report that TiH_2_ powders start to decompose at 550 °C [16], as shown in Equation (1). For compacts sintered at 700 °C, peaks of TiH_2_ disappear. The XRD pattern only has peaks of Ti, Si and C, proving that TiH_2_ decomposes into Ti completely, and no new phase is generated at this temperature, as displayed in Figure 2. These changes are consistent with the data in Figure 1. There are small amounts of TiC (JCPDS: 89-3828) and Ti_5_Si_3_ (JCPDS: 29-1362) phases appearing after sintering at 800 °C for 1 h, which confirms that when the sintering temperature is higher than 700 °C, C and Si around Ti begin to react with each other, as described in Equations (2) and (3), and TiC and Ti_5_Si_3_ are formed as intermediate phases. Beyond 800 °C, the contents of Ti_5_Si_3_ and TiC gradually increase with the rising sintering temperatures. The final phase Ti_3_SiC_2_ (JCPDS: 48-1826) appears in the XRD pattern after sintering at 1200 °C for 1 h, indicating that the formation of Ti_3_SiC_2_ is the result of the reactions among TiC, Ti_5_Si_3_ and unreacted Si as Equation (4) when the sintering temperature is higher than 1100 °C. After sintering compacts at 1300 °C for 1 h, the intensity of peaks indexed to Ti_3_SiC_2_ becomes stronger, while that of TiC and Ti_5_Si_3_ becomes weaker, as shown in Figure 2. The final sintering step is at 1350 °C for 3 h, and the Ti_3_SiC_2_ phase was completely synthesized. Moreover, Si evaporated in the high sintering temperatures, and the reaction can be described as Equation (5).

TiH_2_ → Ti + H_2_ (500–700 °C) (1)

Ti + C → TiC (>700 °C)
(2)

5Ti + 3Si → Ti_5_Si_3_ (>700 °C) (3)

10TiC + Ti_5_Si_3_ + 2Si → 5Ti_3_SiC_2_ (>1100 °C) (4)

Si(s) → Si(g)
(5)

The relative volume percentage of Ti_3_SiC_2_ in the synthesized products can be calculated by the calibrated standard addition method [20] according to the following formula:

(6) $$V_{\mathrm{TSC}}=\frac{I_{\mathrm{TSC}}/I_{TC}}{1.95+I_{TC}/I_{TSC}}$$

*V*_TSC_ is the volume percentage of the Ti_3_SiC_2_ phase. *I*_TSC_/*I*_TC_ is the integrated diffraction intensity ration of Ti_3_SiC_2_ to the TiC main peaks. The volume content of Ti_3_SiC_2_ calculated according to Equation (6) is 99.4 vol %.

The reaction process for the synthesis of Ti_3_SiC_2_ has been studied extensively. Barsoum et al. [21] reported that the intermediate phases are TiC_x_ and Ti_5_Si_3_C_x_ using Ti/SiC/C powders as the raw materials; while Yang et al. [22] observed only the intermediate phase Ti_5_Si_3_ during the reaction process among Ti/Si/TiC for the fabrication of Ti_3_SiC_2_. Sato et al. [23] investigated the formation sequence of Ti_3_SiC_2_ from Ti/Si/C powders, showing that firstly, TiC was produced by the reaction between Ti and graphite; secondly, eutectic Ti-Si liquid appeared around the eutectic temperature (1603 K); finally, Ti_3_SiC_2_ grew due to the existence of both the eutectic liquid phase and TiC. Zhang et al. [24] reported Ti_3_SiC_2_ mainly formed from the reaction between Ti_5_Si_3_C_x_, TiC_x_, TiSi_2_ and graphite at 1400 to 1500 °C. Klemm et al. [25] found intermediate phases of Ti_5_Si_3_ and TiC during the synthesis of Ti_3_SiC_2_ by Ti/Si/C/SiC powders, and Ti_5_Si_3_ and TiC were retained as the impurities in Ti_3_SiC_2_. Although the specific reaction steps of synthesizing Ti_3_SiC_2_ are different in terms of various raw materials, Ti_5_Si_3_ and TiC are usually accepted as intermediate phases due to a coherent Ti_5_Si_3_/TiC interfacial structure with low strain energy [26].

### 3.3. Pore Evolvement and Formation Procedure

**Figure 3:**
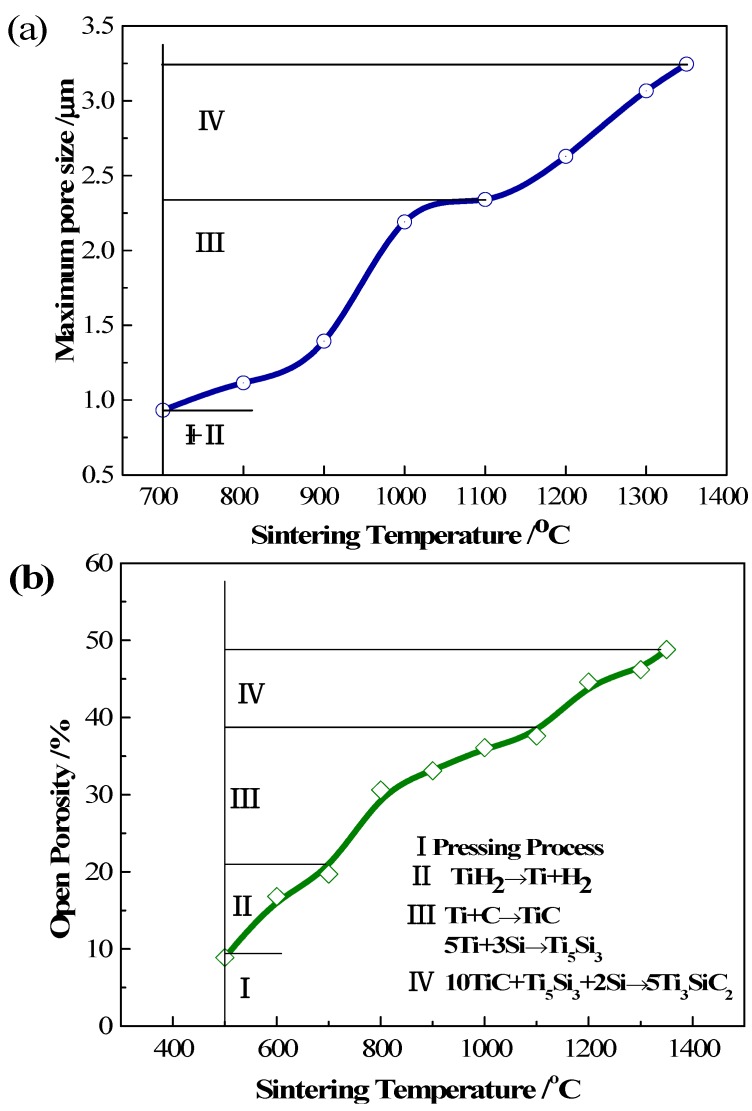
(**a**) The maximum pore size and (**b**) open porosity as a function of the sintering temperature.

Figure 3a displays the variations of the maximum pore size as a function of the sintering temperature. It is evident that the maximum pore size increases with raising the sintering temperature gently. However, when we scrutinize the variations of pore size, we can observe that the changes are different with the gradual increase of sintering temperature with the interval of 100 °C. When the sintering temperature is below 700 °C, the only reaction is the dehydrogenation of TiH_2_ (Equation (1)). Elemental powders of Ti, Si and graphite do not react with each other. Pores in the compacts derive from the interstitial holes formed in the cold press and the room left by the dehydrogenation of TiH_2_. At this stage, the maximum pore size is only about 0.9 μm after sintering at 700 °C for 1 h. Beyond 700 °C, the maximum pore size has an obvious increase and is up to 2.3 μm after sintering at 1100 °C for 1 h before the final phase of Ti_3_SiC_2_ appears. The increase of the maximum pore size between 900 and 1000 °C may be due to the dramatic reactions among the elemental powders. The maximum pore size of the sample reaches 3.2 μm after Ti_3_SiC_2_ is fully synthesized.

The open porosity of compacts also increases gradually with raising the sintering temperatures, as shown in Figure 3b. When the sintering temperature is below 500 °C, no reaction happened. Pores in compacts come from the interstitial holes. The open porosity of the green, pressed compact is only 8.9%. After the compacts are sintered at 700 °C, the open porosity increases to 19.7% accompanying the dehydrogenation of TiH_2_. Then, the open porosity gradually rises to 37.8% after sintering at 1100 °C for 1 h, considering that elemental powders react and intermediate phases, Ti_5_Si_3_ and TiC, are formed. Finally, the open porosity reaches to 46.8% after Ti_3_SiC_2_ is completely synthesized. The overall porosity of porous Ti_3_SiC_2_ measured by the Archimedes method is 50.2%.

**Figure 4:**
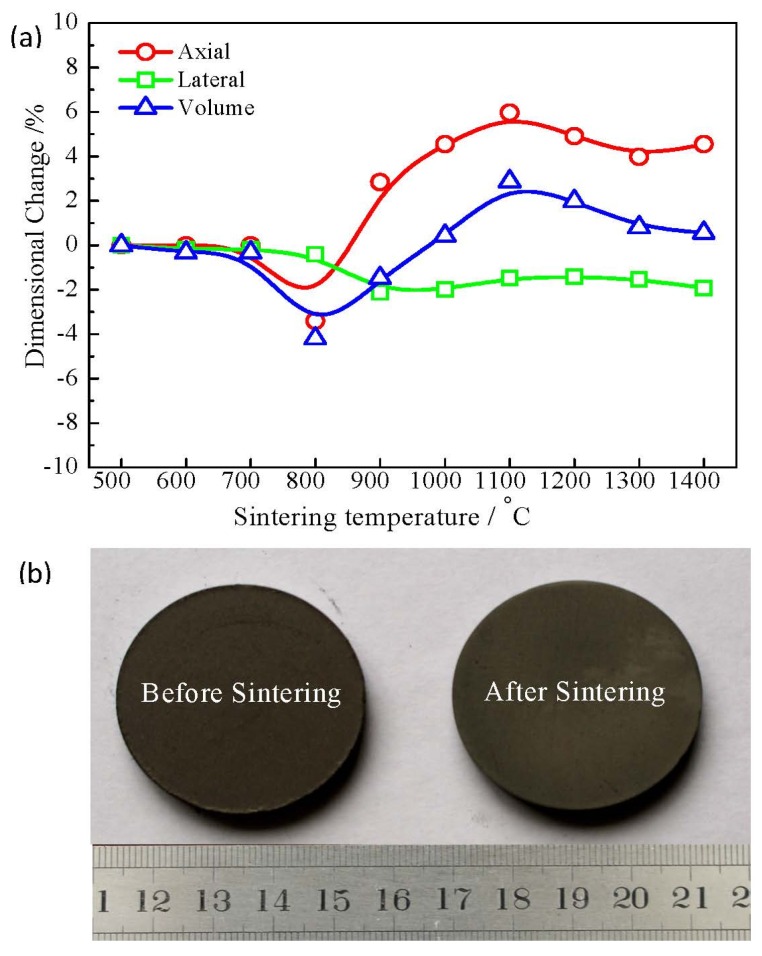
(**a**) Axial, lateral and volume size changes of the compacts sintered at different temperatures; (**b**) Photos of porous compacts before and after sintering

To understand the pore formation procedure of porous Ti_3_SiC_2_ by reactive synthesis, pores produced in every procedure were calculated. Firstly, pores come from the inter-particle holes during the pressing procedure. Five-point-three grams of mixed powders were used to press a green compact with dimensions of ~φ 30 mm × ~3 mm. According to the weight ratios and density of the elemental powders as shown in Table 1, the occupied volumes for TiH_2_, Si and C powders are 46.2%, 17.4% and 12.7% in the pressed compact, respectively. Therefore, the overall porosity of the green compact is 23.7%, and the open porosity is measured to be 8.9%. Table 2 shows the volume expansion/contraction and the porosity produced in the phase transformation process. The volume changes ∆*V* were calculated based on the densities of the reactants and the resultants. The negative value of ∆*V* means volume contraction. Therefore, besides the pores produced in the pressing process, the pores formed accompany the phase transformation procedure. Secondly, pores were produced during the decomposing of the TiH_2_ process. The volume of Ti is smaller than that of TiH_2_, as shown in Table 2, showing 17.3% volume contraction for Reaction (1); while the volume of the compact is nearly constant with the green compact, as shown in Figure 4a, leading to 8.0% pores. Thirdly, pores were formed along with Reactions (2) and (3). Reactions (2) and (3) have volume contractions of 23.4% and 15.6%, respectively, producing 9.0% and 3.4% pores in this process. Finally, the intermediate phases TiC and Ti_5_Si_3_ reacted with Si. This reaction also has a volume contraction of 2.0% and produces 1.1% pores. At the high sintering temperature, the evaporation of Si also leads 2.9% pores. The synthesized Ti_3_SiC_2_ phase can be calculated to occupy 52.1% of the volume, indicating that 47.9% pores remained in the sintered compacts. The total pores produced in the above four procedures are 48.1% (23.7% + 8.0% + 9.0% + 3.4% + 1.1% + 2.9%), which is basically the same as the value calculated by the volume of Ti_3_SiC_2_. The calculated results are also consistent with the experiment results within the measurement uncertainties. The initial pore in the green compact will gradually connect with the pore produced during the reaction process, and the closed pores between the inter particles almost transformed the open pores completely with elevating of the sintering temperatures.

From Table 2, it can be clearly seen that all reactions cause volume contractions. However, the exterior volume of the compacts only has 1% expansion after being sintered completely, as shown in Figure 4a. There is a small contraction for the compacts in the lateral direction due to the volume contraction of the materials; while the axial size becomes slightly larger due to the spring-back happening after the pressing step. We can see that the shape and size of the sintered sample are close to that of the green compact, as shown in Figure 4b. Obviously, we can confirm that the volume of the geometric exterior of the sintered compact is nearly the same as that of the green compact, while the volume of the interior structure is contracted, which can be explained as the formation of pores accompanying the phase transformation process in the compact. Therefore, the pore formation mechanism is mainly due to the phase transformation. The ratios of pore produced in the four reactions are listed in Table 2.

### 3.4. Microstructure Development of Porous Ti_3_SiC_2_

**Figure 5:**
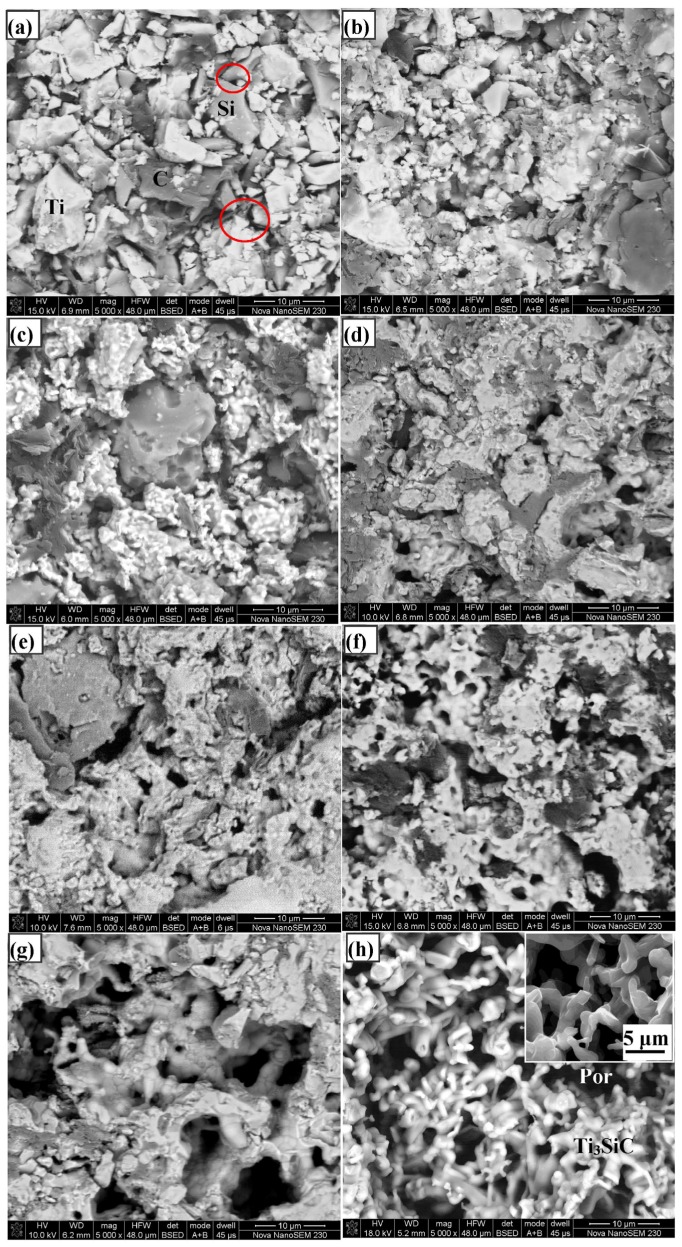
Corresponding back-scattered electron micrographs of the samples sintered at: (**a**) 700 °C; (**b**) 800 °C; (**c**) 900 °C; (**d**) 1000 °C; (**e**) 1100 °C; (**f**) 1200 °C; (**g**) 1300 °C for 1 h; and (**h**) 1350 for 3 h; the inset is the second electron image.

**Figure 6:**
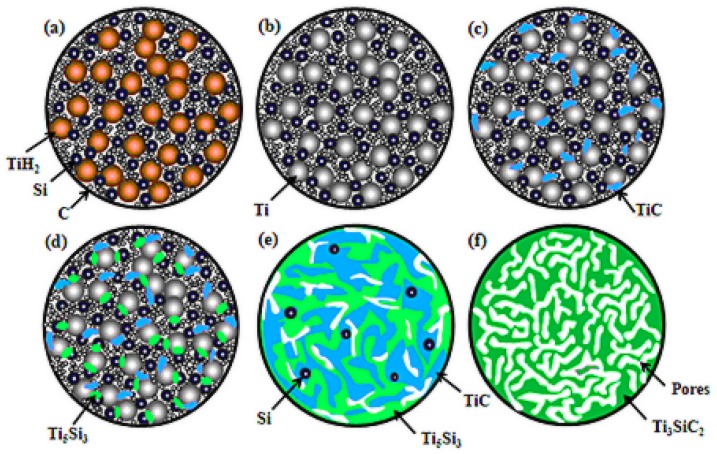
The schematic diagram of the reaction and pore evolution procedure in the sintering process. (**a**) A green compact is composed of TiH_2_, Si and C; (**b**) TiH_2_ decomposed into Ti; (**c**) TiC began to generate; (**d**) Ti_5_Si_3_ began to generate; (**e**) Intermediate phases of TiC and Ti_5_Si_3_ were completely produced; (**f**) Porous Ti_3_SiC_2_ were fabricated.

The pore morphology evolution of porous compacts was observed by SEM. Figure 5 shows the comparisons of the pore morphology of compact discs sintered at different temperatures. Figure 5a is the image of the disc sintered at 700 °C, showing that the elemental particles are even, and clear boundaries exist between independent particles. The adequate contacts between the three kinds of raw powders are guaranteed based on this particle distribution feature, which is beneficial to the solid diffusion reaction of Ti, Si and C. This clearance porosity of green compacts was measured to be about 23.4% and will remain in the sintered compacts, which is the first step in which pores formed, as shown in Figure 3b, and the contribution ratio is about 48.9% for the overall porosity. After the compacts are sintered at 800 °C for 1 h, it is noticeable that the compacts consist of some new pores that are different from the interstitial pores, as shown in Figure 5b. Accompanying the XRD pattern, we can confirm that Ti, C and Si diffuse and react as Equations (2) and (3), and the volume contraction produces pores. It can also be clearly seen from the images (Figure 5c–e) that the pore size and porosity increase with the rising sintering temperature, which is consistent with the test results as shown in Figure 3. Beyond 1100 °C, the framework gradually formed following the final phase transformation, and this process also generated pores. Finally, after sintering at 1350 °C for 3 h, we can find that the material contrast (Figure 5h) is uniform (note that the dark regions are pores), confirming that the compact disc consists of only the Ti_3_SiC_2_ phase, which is consistent with the XRD pattern (Figure 2). The inset of Figure 5h is the secondary electron image of the synthesized porous Ti_3_SiC_2_, and we can find that the pore surface is smooth and the pore size is uniform. The raw particles in the green compact (Figure 5a) have completely evolved into an integral skeleton of Ti_3_SiC_2_ (Figure 5h) after reactive synthesis among the Ti, Si and C elements. Unlike the coarse reactant particles (Figure 5a), the resultant skeleton had a smooth surface, and a large number of interconnected pores was generated along the resultant grains. From the pore size and morphology evolvement law given above, it can be noted that pores are formed in four different steps in our reactive synthesis of porous Ti_3_SiC_2_ ceramics, as illustrated in Figure 6. They are inter-particle pores formed during the pressing procedure; pores produced during the decomposing of TiH_2_; pores formed along with Equations (2) and (3) due to the volume contraction; and pores produced due to the phase transformation of TiC and Ti_5_Si_3_ to the final phase Ti_3_SiC_2_.

## 4. Conclusions

Porous Ti_3_SiC_2_ with purity of 99.4 vol % is fabricated successfully through reactive sintering of TiH_2_, Si and graphite powders. Our results show that pores in porous Ti_3_SiC_2_ mainly derive from the following four aspects: (1) pores formed during the cold-pressing procedure; (2) pores left by the decomposition of TiH_2_; (3) pores formed accompanying the generation of the Ti_5_Si_3_ and TiC intermediate phases by reactions between Ti and Si, Ti and C; (4) pores produced during the final phase Ti_3_SiC_2_ synthesized through the reaction of TiC, Ti_5_Si_3_ and Si. The porosities formed in the four procedures are 23.7%, 8.0%, 12.4% and 4.0%, and the pores are mainly from the phase transformation process.

## Figures and Tables

**Table 1:** The volume ratios of the raw materials.

Raw Material	Atomic Weight	Ratio (wt %)	Density (g/cm^3^)	Occupied Volume (%)
TiH_2_	49.9	72.1	3.91	46.2
Si	28.1	16.3	2.34	17.4
C	12.0	11.6	2.25	12.7

**Table 2:** The volume and pore changes after the reactions.

Reaction	Resultant	Occupied Volume (%)	Density (g/cm^3^)	Volume Change (%)	Formed Pore (%)	Contribution Rate (%)
(1)	Ti	38.2	4.54	−17.3	8.0	16.7
(2)	TiC	29.3	4.93	−23.4	9.0	18.8
(3)	Ti_5_Si_3_	18.1	4.32	−15.6	3.4	7.1
(4)	Ti_3_SiC_2_	52.1	4.53	−2.0	1.1	2.3
(5)	Si(g)	0	2.34	−100	2.9	6.1
